# Supplementary material for: Synthesis and In Vitro Evaluation of Gold Nanoparticles Functionalized with Thiol Ligands for Robust Radiolabeling with 99mTc
Source: Nanomaterials (Basel). 2021 Sep 15;11(9):2406. doi: 10.3390/nano11092406 (PMC8471789; doi:10.3390/nano11092406)
Supplement: Supplementary file 1 [file nanomaterials-11-02406-s001.zip › nanomaterials-1358088-supplementary .pdf]

Supplementary Materials

Synthesis and In Vitro Evaluation of Gold Nanoparticles Functionalized with Thiol Ligands for Robust Radiolabeling with ^{99m}Tc

Adamantia Apostolopoulou [1,2], Aristeidis Chiotellis [1], Evangelia-Alexandra Salvanou [1], Konstantina Makrypidi [1], Charalampos Tsoukalas [1], Fotis Kapiris [1], Maria Paravatou-Petsotas [1], Minas Papadopoulos [1], Ioannis C. Pirmettis [1], Przemysław Koźmiński [3] and Penelope Bouziotis [1,*]

1 National Center for Scientific Research "Demokritos", Institute of Nuclear & Radiological Sciences & Technology, Energy & Safety, 15341 Agia Paraskevi, Athens, Greece; madoapostol@gmail.com (A.A.); achiotel@rrp.demokritos.gr (A.C.); salvanou@rrp.demokritos.gr (E.-A.S.); kmakripidi@gmail.com (K.M.); ctsoukal@rrp.demokritos.gr (C.T.); fotiskp@gmail.com (F.K.); mparavatou@rrp.demokritos.gr (M.P.-P.); mspap@rrp.demokritos.gr (M.P.); ipirme@rrp.demokritos.gr (I.C.P.); bouzioti@rrp.demokritos.gr (P.B.)

2 Department of Pharmacy, National and Kapodistrian University of Athens, Panepistimiopolis Zographou, 15771 Athens, Greece

3 Centre of Radiochemistry and Nuclear Chemistry, Institute of Nuclear Chemistry and Technology, Dorod-na 16 Str., 03-195 Warsaw, Poland; p.kozminski@ichtj.waw.pl

* Correspondence: Correspondence: bouzioti@rrp.demokritos.gr; Tel.: +30-2106503687

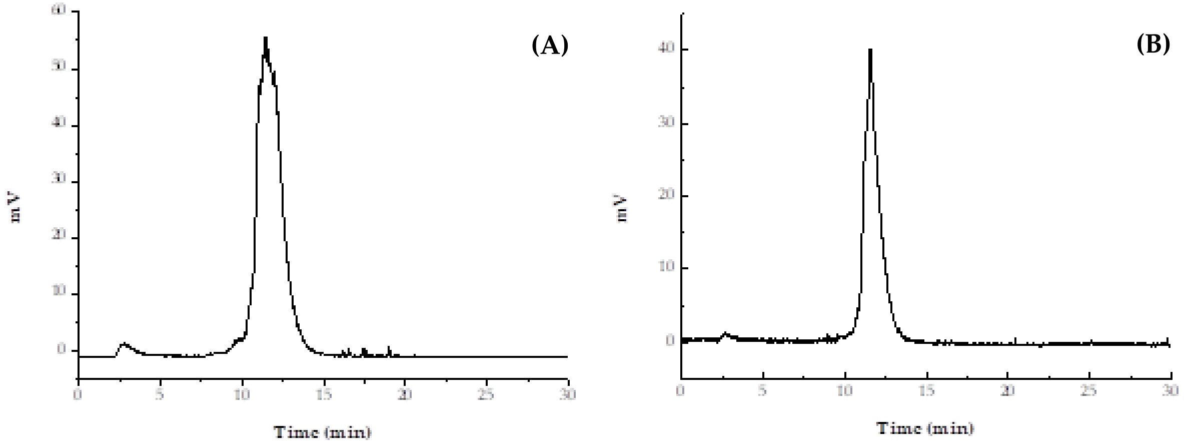

Figure S1. HPLC Diagrams representing the radiochemical purity of $[^{99m}Tc]Tc\text{-}Au^{(2)}L_1$ at (A) 1 h and (B) 24 h post-incubation

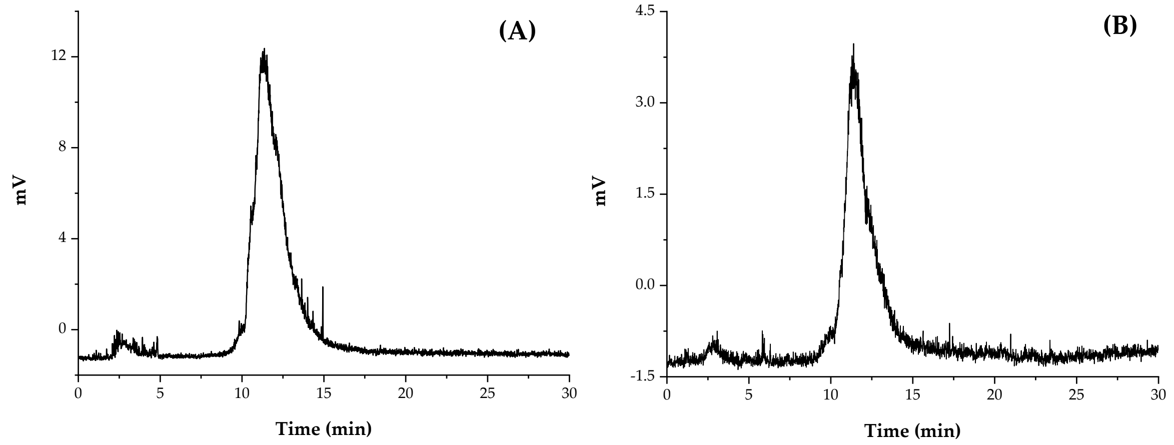

Figure S2. HPLC Diagrams representing the *in vitro* stability of $[^{99m}Tc]Tc\text{-}Au^{(2)}L_1$ in cysteine solution at (A) 1 h and (B) 24 h post-incubation

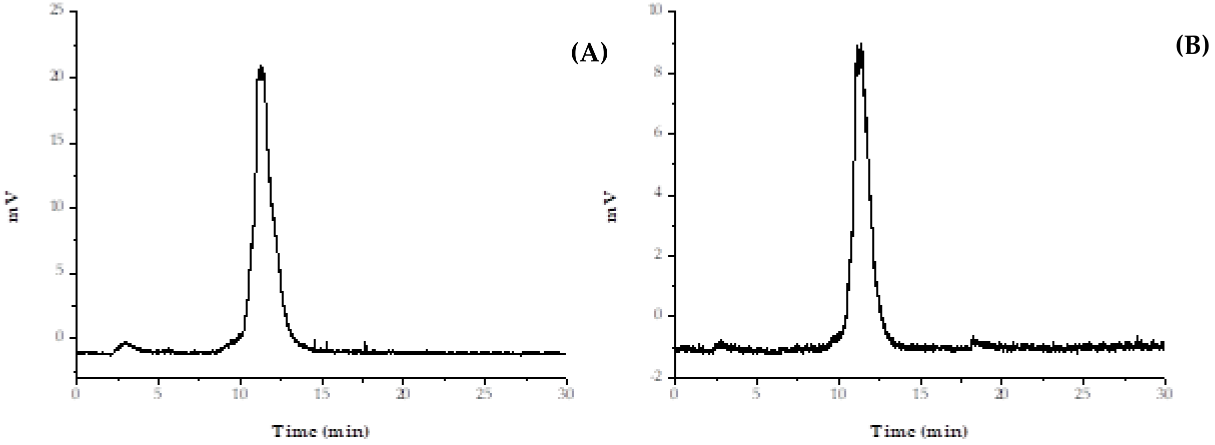

Figure S3. HPLC Diagrams representing the *in vitro* stability of $[^{99m}Tc]Tc\text{-}Au^{(2)}L_1$ in histidine solution at (A) 1 h and (B) 24 h post-incubation

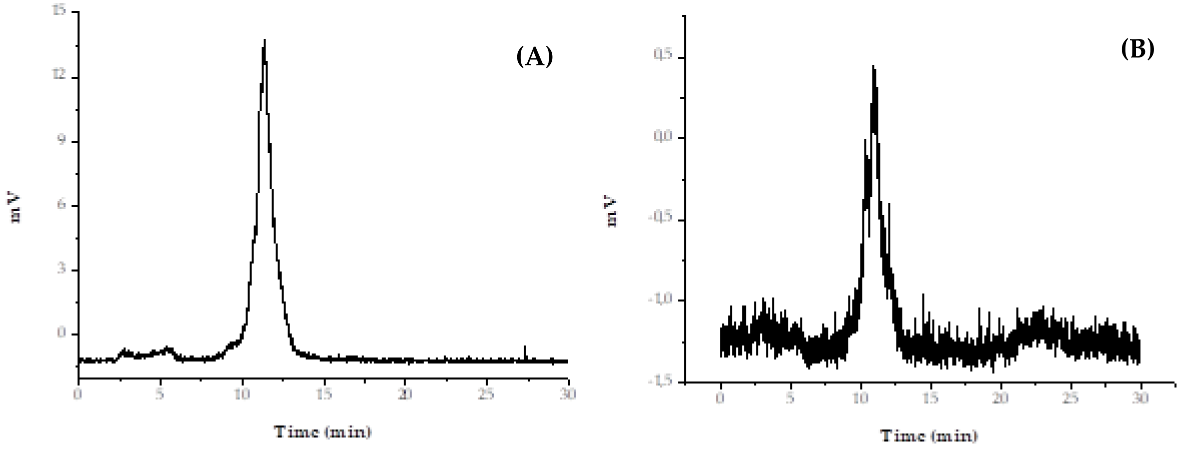

Figure S4. HPLC Diagrams representing the *in vitro* stability of $[^{99m}Tc]Tc\text{-}Au^{(2)}L_1$ in human serum at (A) 1 h and (B) 24 h post-incubation

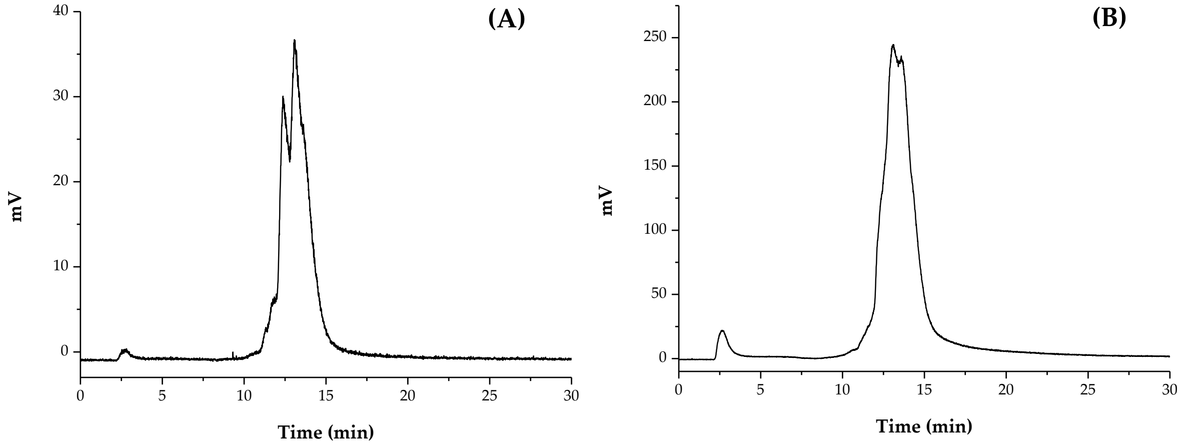

Figure 5. HPLC Diagrams representing the radiochemical purity of $[^{99m}Tc]Tc\text{-}Au^{(2)}L_2$ at (A) 1 h and (B) 24 h post-incubation.

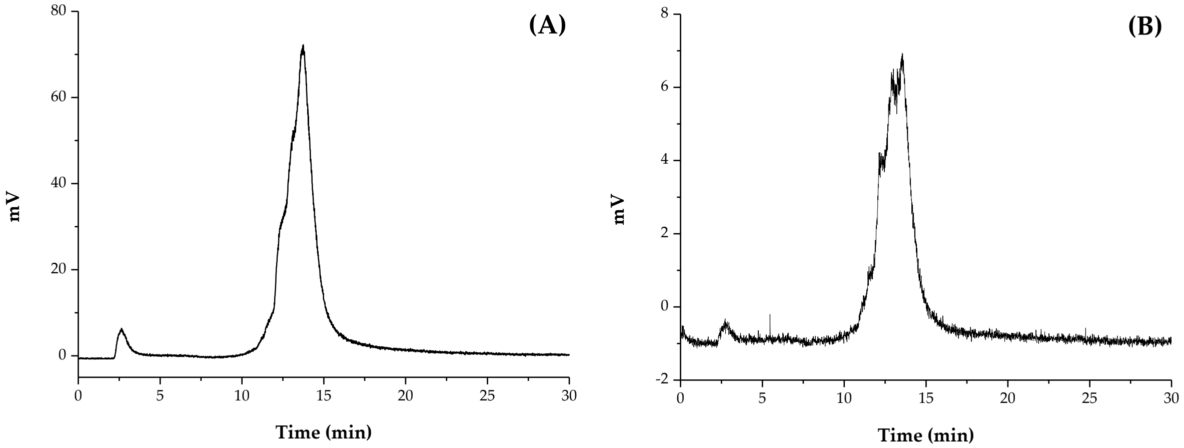

Figure S6. HPLC Diagrams representing the *in vitro* stability of $[^{99m}Tc]Tc\text{-}Au^{(2)}L_2$ in cysteine solution at (A) 1 h and (B) 24 h post-incubation

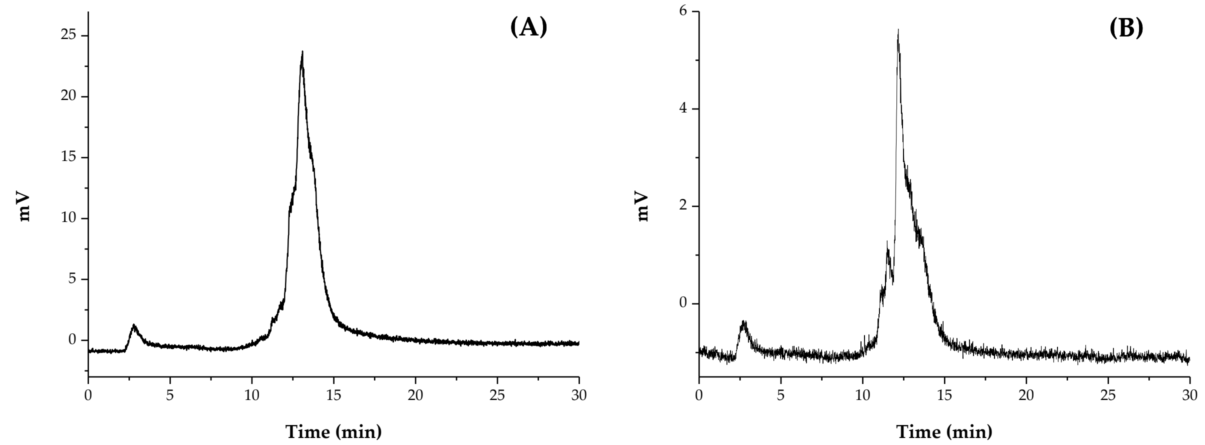

Figure S7. HPLC Diagrams representing the *in vitro* stability of [^{99m}Tc]Tc-Au$^{(2)}$L$_2$ in histidine solution at (A) 1 h and (B) 24 h post-incubation

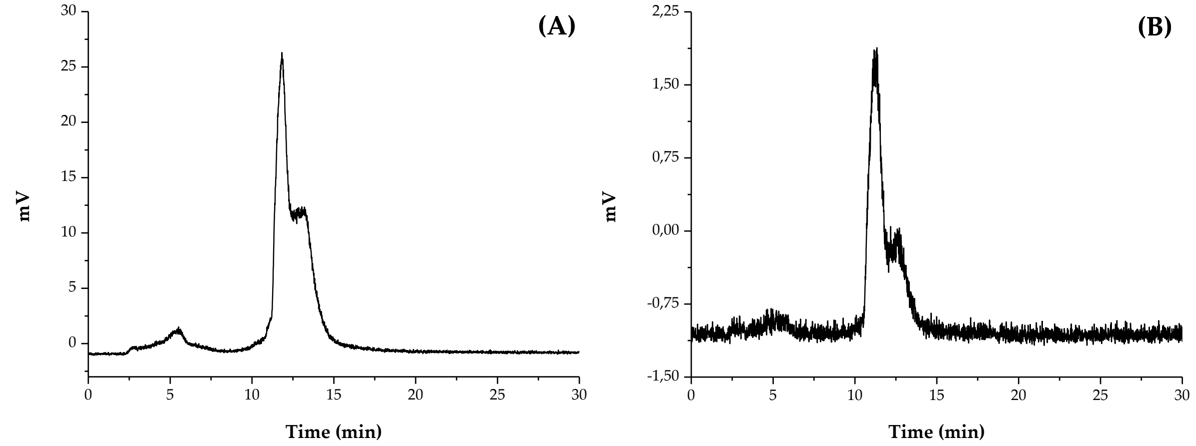

Figure S8. HPLC Diagrams representing the *in vitro* stability of [^{99m}Tc]Tc-Au$^{(2)}$L$_2$ in human serum at (A) 1 h and (B) 24 h post-incubation

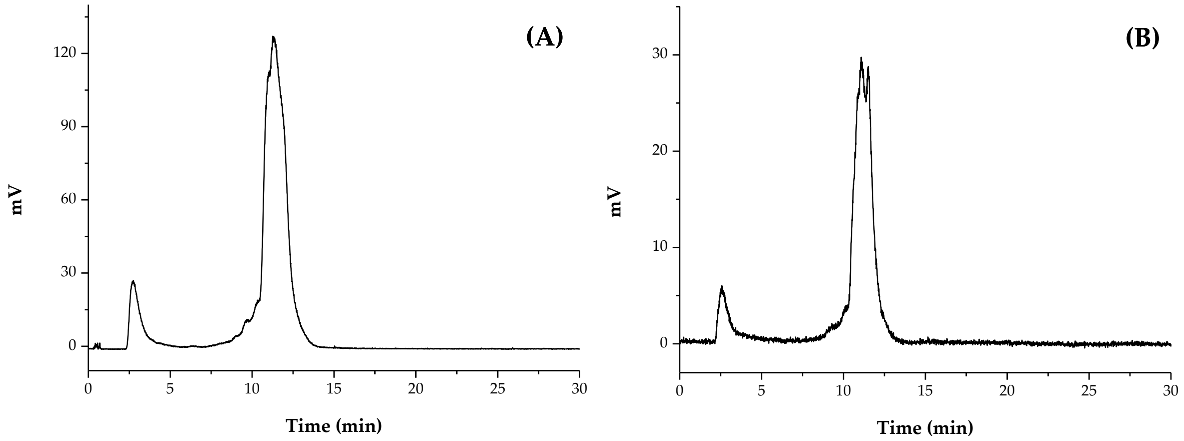

Figure S9. HPLC Diagrams representing the radiochemical purity of [^{99m}Tc]Tc-Au$^{(20)}$L$_1$ at (A) 1 h and (B) 24 h post-incubation

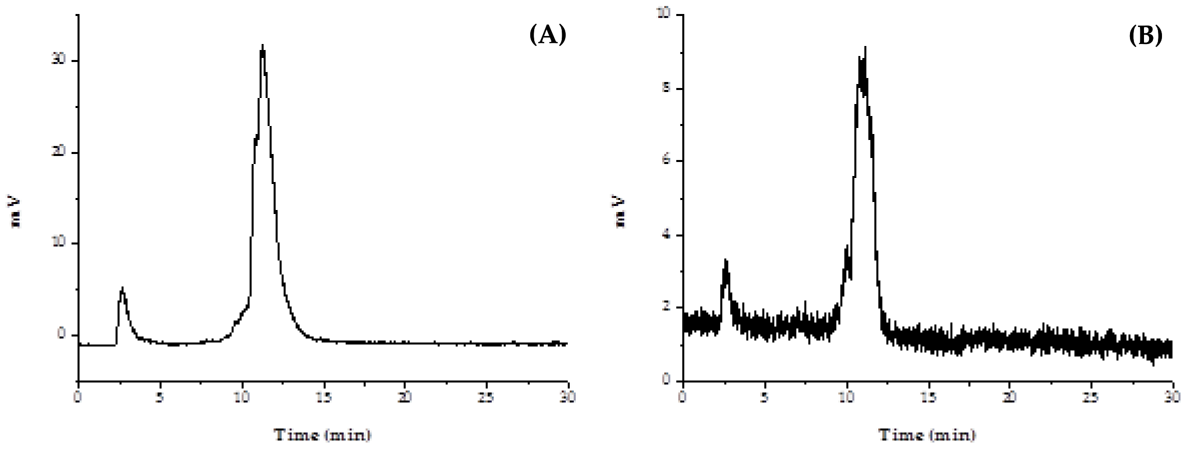

Figure S10. HPLC Diagrams representing the *in vitro* stability of [^{99m}Tc]Tc-Au$^{(20)}$L$_1$ in cysteine solution at (A) 1 h and (B) 24 h post-incubation

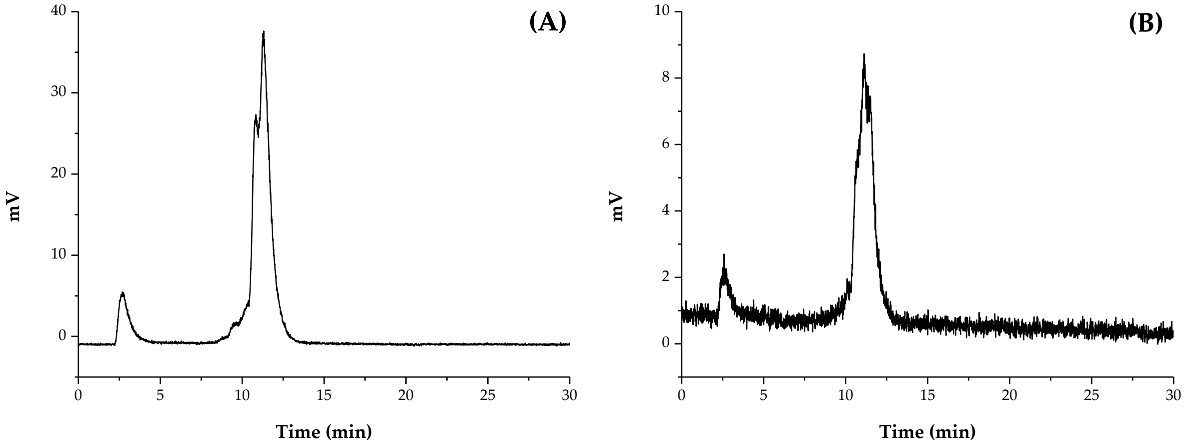

Figure S11. HPLC Diagrams representing the *in vitro* stability of [^{99m}Tc]Tc-Au$^{(20)}$L$_1$ in histidine solution at (A) 1 h and (B) 24 h post-incubation

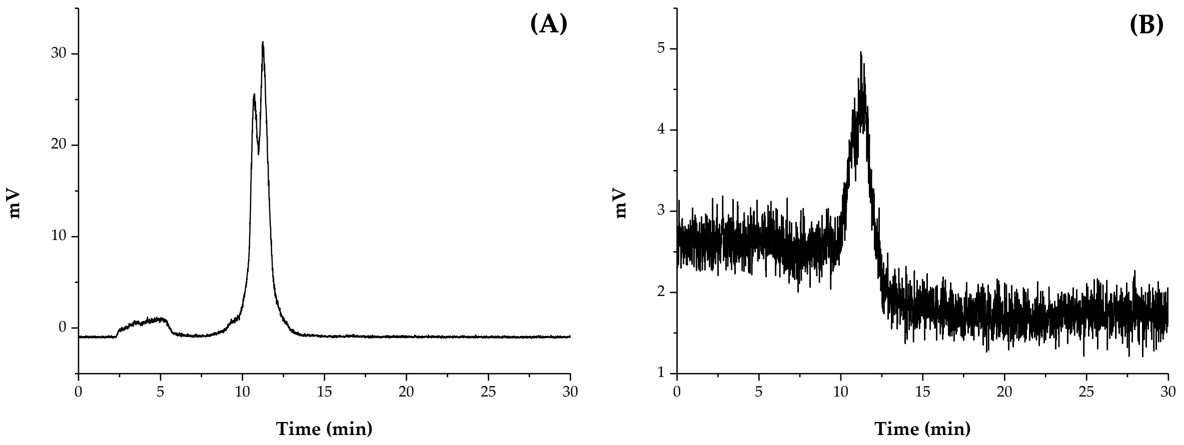

Figure S12. HPLC Diagrams representing the *in vitro* stability of [^{99m}Tc]Tc-Au$^{(20)}$L$_1$ in human serum at (A) 1 h and (B) 24 h post-incubation

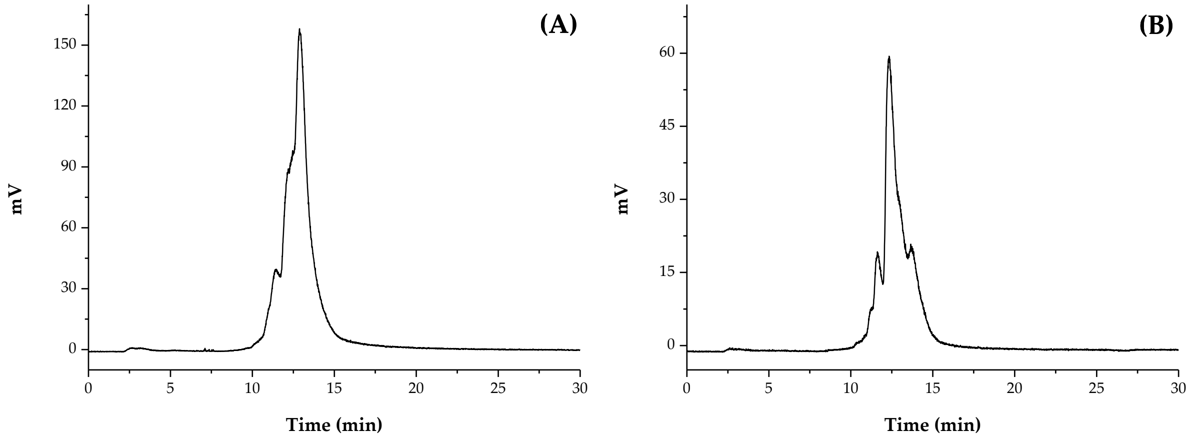

Figure S13. HPLC Diagrams representing the radiochemical purity of [^{99m}Tc]Tc-Au$^{(20)}$L$_2$ at (A) 1 h and (B) 24 h post-incubation

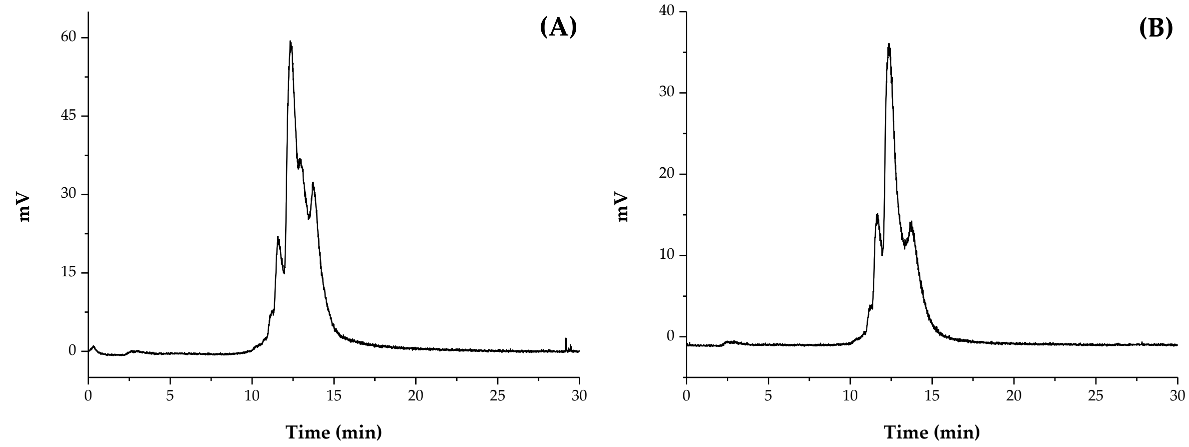

Figure S14. HPLC Diagrams representing the *in vitro* stability of [^{99m}Tc]Tc-Au$^{(20)}$L$_2$ in cysteine solution at (A) 1 h and (B) 24 h post-incubation

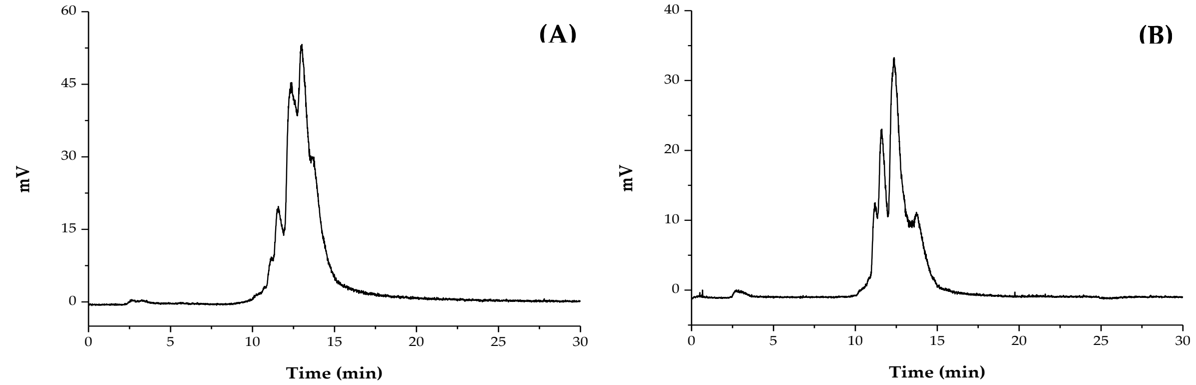

Figure S15. HPLC Diagrams representing the *in vitro* stability of [^{99m}Tc]Tc-Au$^{(20)}$L$_2$ in histidine solution at (A) 1 h and (B) 24 h post-incubation

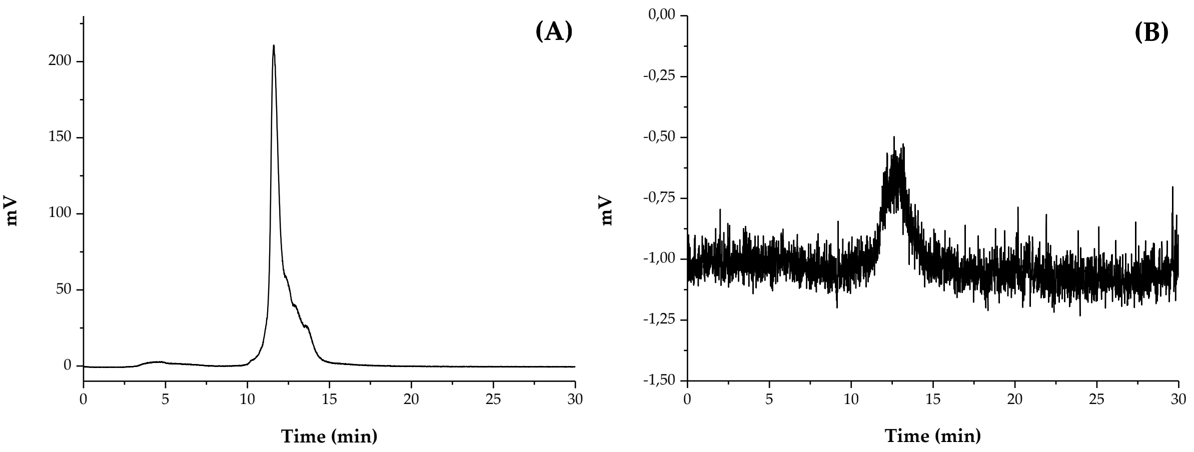

Figure S16. HPLC Diagrams representing the *in vitro* stability of [^{99m}Tc]Tc-Au$^{(20)}$L$_2$ in histidine solution at (A) 1 h and (B) 24 h post-incubation

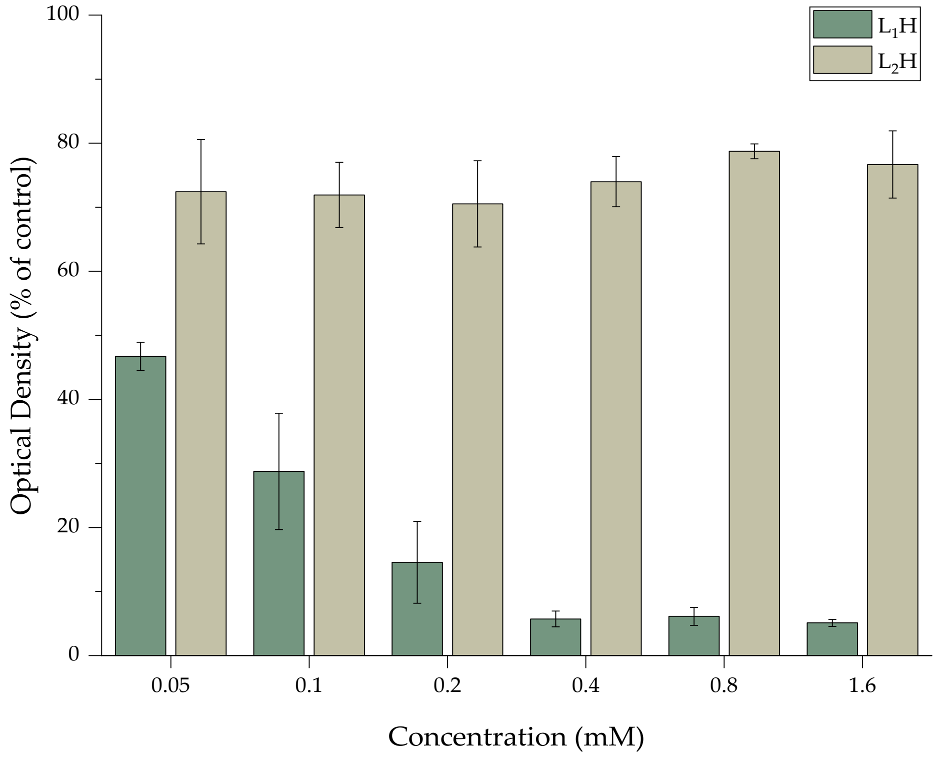

Figure S17. MTT Assay of 4T1 cells treated with different concentrations of L_1H and L_2H. The tested ligand concentrations correspond to the concentrations of the AuNPs used in the MTT Assay.
